# Tribotronic Enhanced Photoresponsivity of a MoS_2_ Phototransistor

**DOI:** 10.1002/advs.201500419

**Published:** 2016-02-18

**Authors:** Yaokun Pang, Fei Xue, Longfei Wang, Jian Chen, Jianjun Luo, Tao Jiang, Chi Zhang, Zhong Lin Wang

**Affiliations:** ^1^ Beijing Institute of Nanoenergy and NanosystemsChinese Academy of SciencesNational Center for Nanoscience and TechnologyBeijing100083P. R. China; ^2^ School of Material Science and EngineeringGeorgia Institute of TechnologyAtlantaGA30332USA

**Keywords:** MoS_2_, photoresponsivity, phototransistor, triboelectric nanogenerator, tribotronics

## Abstract

Molybdenum disulfide (MoS_2_) has attracted a great attention as an excellent 2D material for future optoelectronic devices. Here, a novel MoS_2_ tribotronic phototransistor is developed by a conjunction of a MoS_2_ phototransistor and a triboelectric nanogenerator (TENG) in sliding mode. When an external friction layer produces a relative sliding on the device, the induced positive charges on the back gate of the MoS_2_ phototransistor act as a “gate” to increase the channel conductivity as the traditional back gate voltage does. With the sliding distance increases, the photoresponsivity of the device is drastically enhanced from 221.0 to 727.8 A W^−1^ at the 100 mW cm^−2^ UV excitation intensity and 1 V bias voltage. This work has extended the emerging tribotronics to the field of photodetection based on 2D material, and demonstrated a new way to realize the adjustable photoelectric devices with high photoresponsivity via human interfacing.

## 1 Introduction

Recently, because of the intriguing electrical,^1^ piezoelectric,^2^, ^3^ and optical^4^, ^5^, ^6^ properties, molybdenum disulfide (MoS_2_) has attracted a great interest for the researchers. As a typical 2D material, the bulk MoS_2_ consists of lamellar S–Mo–S atoms units bonded by weakly van der Waals forces, facilitating cleavage of the crystals using the mechanical exfoliation technique or the liquid‐phase method.^7^, ^8^, ^9^ Bulk MoS_2_ is an indirect‐gap semiconductor with a bandgap of 1.2 eV, while monolayer MoS_2_ is a direct‐gap semiconductor with a bandgap of 1.8 eV.^8^, ^10^, ^11^ Field effect transistors using MoS_2_ as semiconductor channel material have exhibited excellent performances, such as high current ON/OFF ratio (10^7^–10^8^), high electron mobility (≈200 cm^2^ V^−1^ S^−1^) and low sub‐threshold swing (74 mV dec^−1^),^1^, ^12^ which have a wide range of potential practical applications in the field of gas sensor,^13^, ^14^ amplifiers,^15^ and logic circuits.^16^ To date, a series of phototransistors based on multilayer MoS_2_ has been reported with broad spectral response from ultraviolet to infrared and high photoresponsivity (880 A W^−1^) and optical gain (*V*_ds_ = 8 V, *V*_gs_ = −70 V),^4^ indicating that MoS_2_ is a promising building‐block for the optoelectronic devices. However, in order to enhance the photoresponsivity of MoS_2_ phototransistors, a high gate bias or large drain–source bias voltage usually needs to be applied to increase the photocurrent, resulting in extra energy dissipation or the increased dark current. A direct adjusting mechanism is not available for interfacing the external environment and phototransistors.

Since 2012, based on the universally known triboelectrification and electrostatic induction effects, the innovative triboelectric nanogenerator (TENG) has been successfully invented to convert ambient mechanical energy into electricity.^17^, ^18^, ^19^, ^20^, ^21^ The TENG has been extensively utilized in microenergy,^22^, ^23^ macroenergy,^24^, ^25^ active sensors,^26^, ^27^, ^28^, ^29^ and triboelectric‐charge‐controlled devices.^30^, ^31^ In 2014, an interesting new field of tribotronics was introduced, which uses electrostatic potential created by TENG as a “gate” voltage to control charge carrier transport in the semiconductor.^32^ By now, several tribotronic devices have been experimentally demonstrated including logic circuits,^33^ organic LED,^34^ memory,^35^ and phototransistors,^36^ especially smart tactile switch based on MoS_2_ tribotronic transistor.^37^

Here in this work, we developed a novel MoS_2_ tribotronic phototransistor by coupling of a MoS_2_ phototransistor and a sliding mode TENG. The photoresponsivity of the MoS_2_ tribotronic phototransistor can be enhanced considerably by a relative sliding between the bottom friction layer and the device. With the sliding distance increasing, electrons flow from the aluminum electrode on the bottom of the fluorinated ethylene propylene (FEP) film to the source electrode and an inner gate voltage is formed, resulting in the increased Femi level of the semiconducting MoS_2_. Consequently, the energy bands of the MoS_2_ will bend downwards and the barrier height between the electrode and the MoS_2_ will be lowered, resulting in increased separation and transport of the photogenerated carriers under illumination. Under an illumination of 100 mW cm^−2^ excitation intensity and 1 V bias voltage, with the sliding range of 8 mm, the photoresponsivity of the device is drastically enhanced from 221.0 to 727.8 A W^−1^. This work has extended the emerging tribotronics to the field of photodetection based on 2D material, and demonstrated a new way to realize adjustable photoelectric devices with high photoresponsivity via human interfacing.

## 2 Principle and Characteristics

### 2.1 Structure of the MoS_2_ Tribotronic Phototransistor

**Figure 1:**
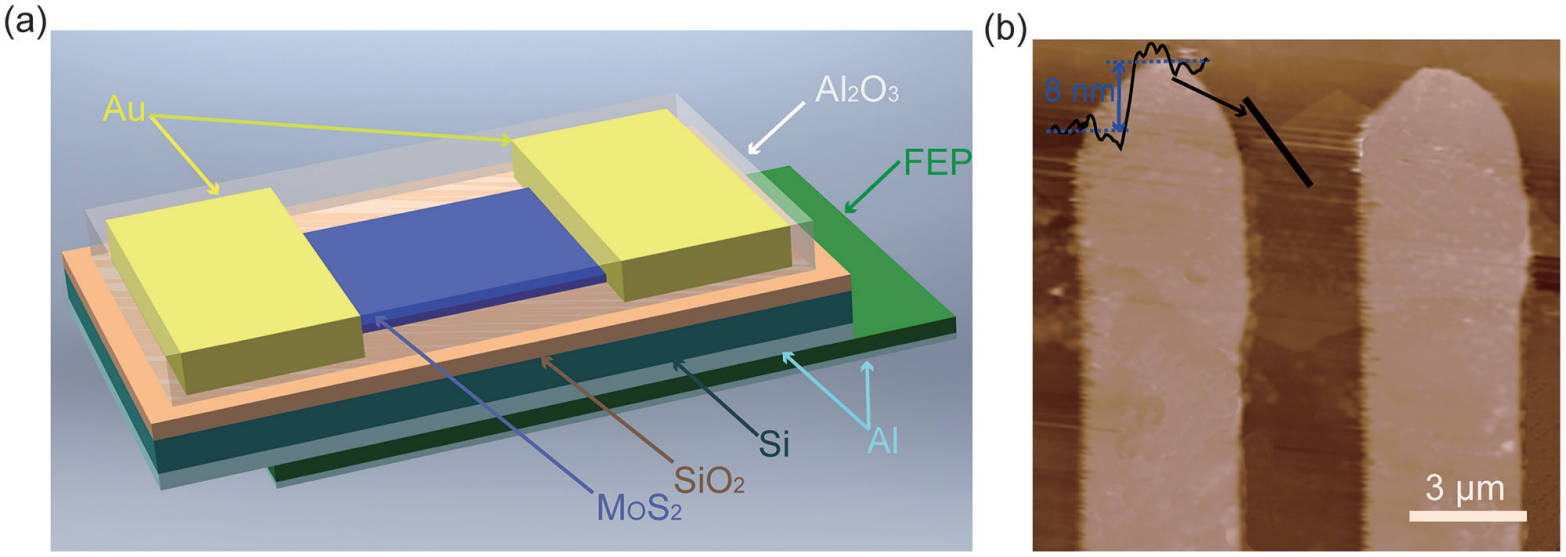
Schematic illustration of a MoS_2_ tribotronic phototransistor. a) Structure of a MoS_2_ tribotronic phototransistor based on a MoS_2_ phototransistor and a triboelectric nanogenerator in sliding mode. b) Atomic force microscope (AFM) image of the MoS_2_ flake used in this device. Inset: Cross‐sectional plot along the black line and the height of the flake is 8 nm.

The basic structure of the MoS_2_ tribotronic phototransistor is composed of a MoS_2_ phototransistor and a TENG in sliding mode, as schematically illustrated in **Figure** [qv: **1**]a. A few‐layer MoS_2_ was prepared on degenerately doped p‐type silicon substrate with 300 nm thick SiO_2_ by using the Scotch tape‐based mechanical exfoliation method. Subsequently, UV lithography and electron beam evaporation were used for the fabrication of source and drain electrodes (5 nm Cr/50 nm Au) on MoS_2_ flakes. An aluminum (Al) film was deposited on the bottom surface of the silicon substrate with a thickness of about 100 nm, serving as a gate electrode. The bottom friction layer, which acts as a free‐sliding layer, consists of a 20 μm thick FEP film (10 mm × 10 mm) attached on a 50 μm thick Al foil. In order to increase the effective surface area and improve the triboelectric charge density, the surface of the FEP was etched by inductively coupled plasma (ICP) (Figure S1, Supporting Information). To keep its stability, the device was packaged with a layer of 30 nm thick Al_2_O_3_ by atomic layer deposition (ALD). Figure 1b shows the atomic force microscopy (AFM) image of the MoS_2_ flake used in this device. The left inset presents that the height of the MoS_2_ flake along the black line is about 8 nm. The conduction channel length and width is 3 μm and 5 μm, respectively.

### 2.2 Characteristics of the MoS_2_ Phototransistor

**Figure 2:**
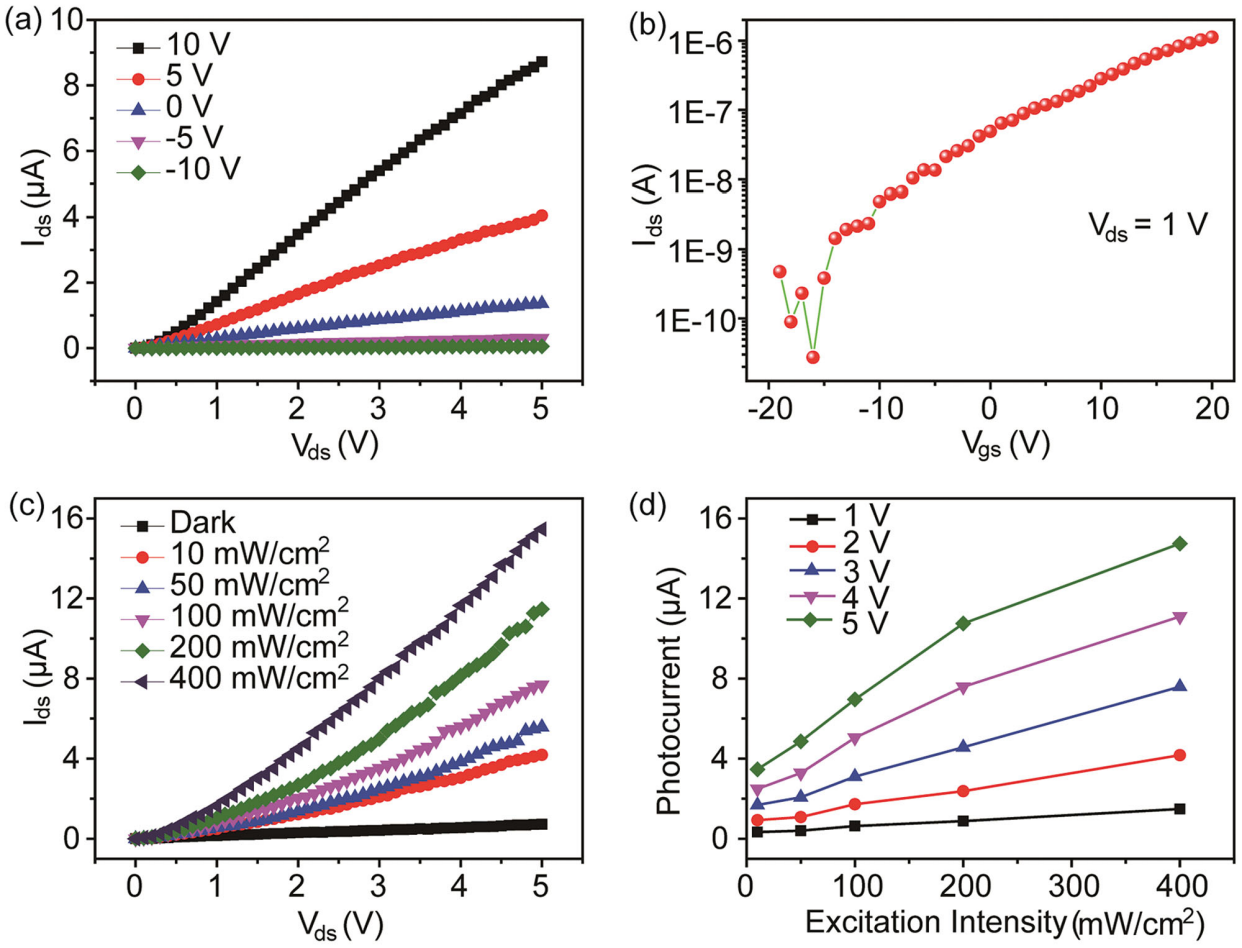
Characteristics of the MoS_2_ phototransistor. a) *I*_ds_–*V*_ds_ output characteristics at different *V*_gs_ without illumination. b) *I*_ds_–*V*_gs_ transfer characteristics without illumination (*V*_ds_ = 1 V). c) *I*_ds_–*V*_ds_ output characteristics at different UV excitation light intensity (*λ* = 365 nm) (*V*_gs_ = 0 V). d) Photocurrent as a function of UV excitation light intensity (*λ* = 365 nm) at different *V*_ds_ (*V*_gs_ = 0 V).

The characteristics of the MoS_2_ phototransistor were measured by a semiconductor parameter analyzer (Keithley 4200) and shielded probe station at room temperature. As shown in **Figure** [qv: **2**]a, the drain–source current (*I*_ds_) varies nearly linearly with *V*_ds_ under different *V*_gs_ from −10 to 10 V. Figure 2b shows the *I*_ds_−*V*_gs_ transfer curves at the drain bias voltage of 1 V, and the current ON/OFF ratio of the device is about 10^3^. The device exhibits the excellent n‐type transistor properties, which are similar to the previous reports of the MoS_2_ transistor.^1^ Without gate voltage, the typical *I*_ds_–*V*_ds_ characteristics of the MoS_2_ phototransistor under the dark and different excitation light intensity (*λ* = 365 nm) are shown in Figure 2c. It is presented that the phototransistor is very sensitive to the excitation light intensity and the drain–source current increases with the increasing illumination. When the illumination increases from dark to 400 mW cm^−2^, the *I*_ds_ increase from 0.72 to 15.46 μA at a drain voltage of 5 V, enhancing about 21 times. The relationships between the photocurrent (*I*_ph_ = *I*_light_−*I*_dark_) and the excitation light intensity under different drain voltages are plotted in Figure 2d, which indicate that the photocurrent increases with the excitation light intensity increasing. The photocurrent in different wavelengths and the response time of the MoS2 phototransistor are shown in Figures S2 and S3 (Supporting Information), respectively. The measured results distinctly demonstrated that the MoS2 has good photosensitive prosperity and can be used as semiconductor channel material in phototransistor.

### 2.3 Characteristics of the MoS_2_ Tribotronic Phototransistor

**Figure 3:**
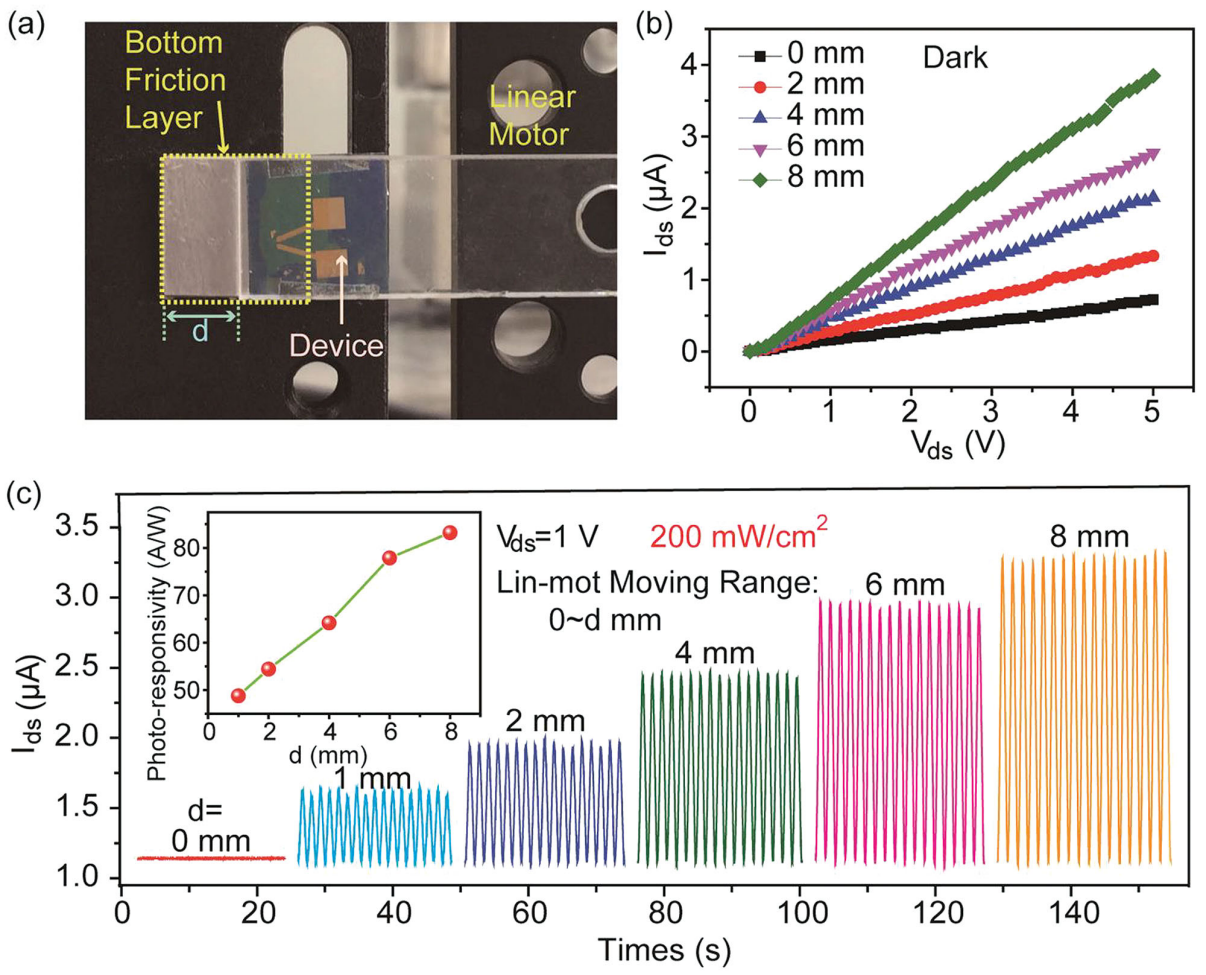
Characteristics of the MoS_2_ tribotronic phototransistor. a) Optical graph of the testing set for the device. The phototransistor is fastened on a linear motor while the bottom friction layer mounted on a fixed bracket. The sliding distance between the bottom friction layer and the device is labeled as *d*. b) *I*_ds_–*V*_ds_ output characteristics as a function of d without illumination. c) *I*_ds_ at different sliding distances with 200 mW cm^−2^ excitation intensity and 1 V bias voltage. The photoresponsivity versus *d* is plotted in the inset.

The characteristics of the MoS_2_ tribotronic phototransistor are investigated independently, as shown in **Figure** [qv: **3**], by applying different sliding distances. The testing set for the device is shown in Figure 3a. The phototransistor is fastened on a linear motor, which can accurately control the sliding distance of the device. The bottom friction layer is supported by an arclic plate mounted on a fixed bracket and the surface of the FEP keeps closely contact with the aluminum film on the bottom of the device. The defined sliding distance between the bottom friction layer and the device is labeled as d. Figure 3b shows the *I*_ds_−*V*_ds_ characteristics of the MoS_2_ tribotronic phototransistor at different sliding distances without illumination and external gate voltage. It is observed that the drain‐source current is increased with the increasing of the sliding distances *d*. The drain–source current for different sliding distances with 200 mW cm^−2^ excitation intensity and 1 V bias voltage are shown in Figure 3c. In the original state, the device is contacted with the whole bottom friction layer and the sliding distance *d* is 0 mm. Driven by the programmed linear motor with a fixed frequency, the device moves back and forth to a certain distance from the original position. With the sliding distance from 0 to 8 mm, the change in drain–source current are uniform and stable, and the drain–source current can increase from 1.05 to 3.24 μA, nearly for three times enhancement. It is demonstrated that the changing of the sliding distance can effectively and stably tune the drain‐source current as well as the photocurrent. The photoresponsivity is defined as *I*_ph_/*P*_ill_, where *P*_ill_ is the illumination power on the phototransistor. The photoresponsivity of the MoS_2_ tribotronic phototransistor versus sliding distance *d* is plotted in the inset of Figure 3c, which is increased from 48.81 to 83.19 A W^−1^ with sliding distance of 8 mm. The generated positive gate voltage corresponding to the different sliding distance is shown in Figure S4 (Supporting Information). It is distinctly illustrated that the TENG can effectively improve the phototransistor and the photoresponsivity. Compared with the output and transfer characteristics of the MoS_2_ phototransistor with external gate voltage source in Figure 2a,b, the characteristics of the MoS_2_ tribotronic phototransistor has been successfully adjusted and enhanced by the sliding electrification, which has the same effect as applying a gate voltage.

### 2.4 Tribotronic Enhanced Photoresponsivity of the MoS_2_ Phototransistor under Different Excitation Intensity

**Figure 4:**
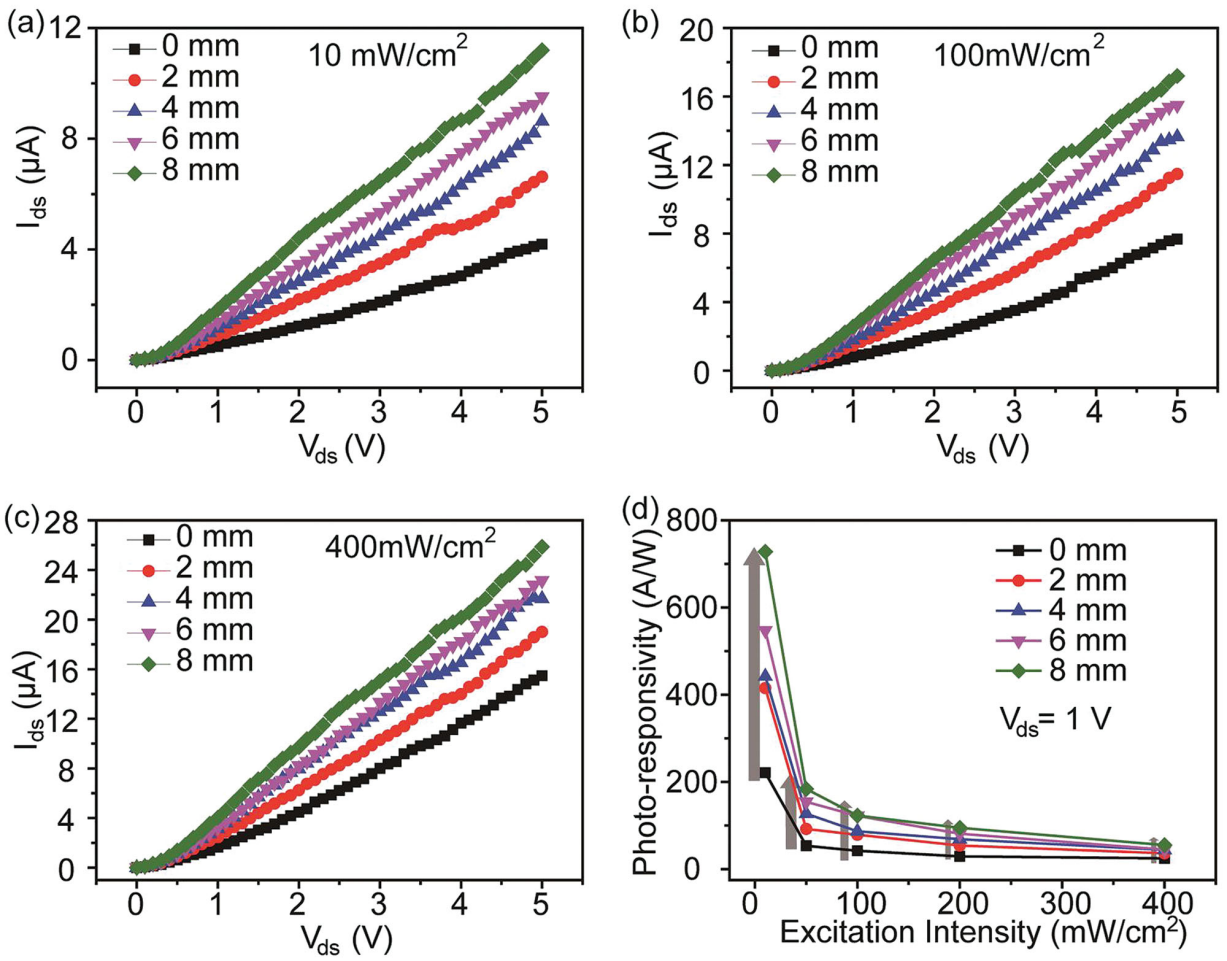
Tribotronic enhanced photoresponsivity of the MoS_2_ phototransistor under different excitation intensity. a–c) *I*_ds_–*V*_ds_ curves with the different excitation intensity of 10, 100, and 400 mW cm^−2^, respectively. d) Photoresponsivity relative to excitation intensity at different sliding distances (*V*_ds_ = 1 V).

*I*_ds_–*V*_ds_ curves with the different excitation intensity of 10, 100, and 400 mW cm^−2^ are shown in **Figure** [qv: **4**]a–c at different sliding distances. The *I*_ds_–*V*_ds_ curves are almost linear and show an increase of drain–source current with the increasing of the sliding distance and excitation intensity. To quantificationally study the relationship between the photoresponsivity and excitation intensity at different sliding distances, the plot of photoresponsivity and the excitation intensity with 1 V drain voltage are interpreted in Figure 4d. Under the 0 mm sliding distance and 10 mW cm^−2^ illumination, the photoresponsivity is 221.03 A W^−1^ with 1 V drain voltage while increasing to 727.87 A W^−1^ with the sliding distance of 8 mm, which demonstrates that the sliding distance plays an important role in tuning the photoresponsivity of the MoS_2_ phototransistor. *I*_ds_–*V*_ds_ curves with the excitation intensity of 50 and 200 mW cm^−2^, and the plot of photoresponsivity and the excitation intensity with different drain voltages are also shown in Figures S5 and S6 (Supporting Information), respectively, in which the MoS_2_ tribotronic phototransistor has similar characteristics. It is noted that the photoresponsivity and enhanced value by triboelectrification both decrease with the increasing of the excitation intensity, owing to the saturation of trap states either in the MoS_2_ or at the interface between the MoS_2_ and substrate.

### 2.5 Principle Discussion on the MoS_2_ Tribotronic Phototransistor

**Figure 5:**
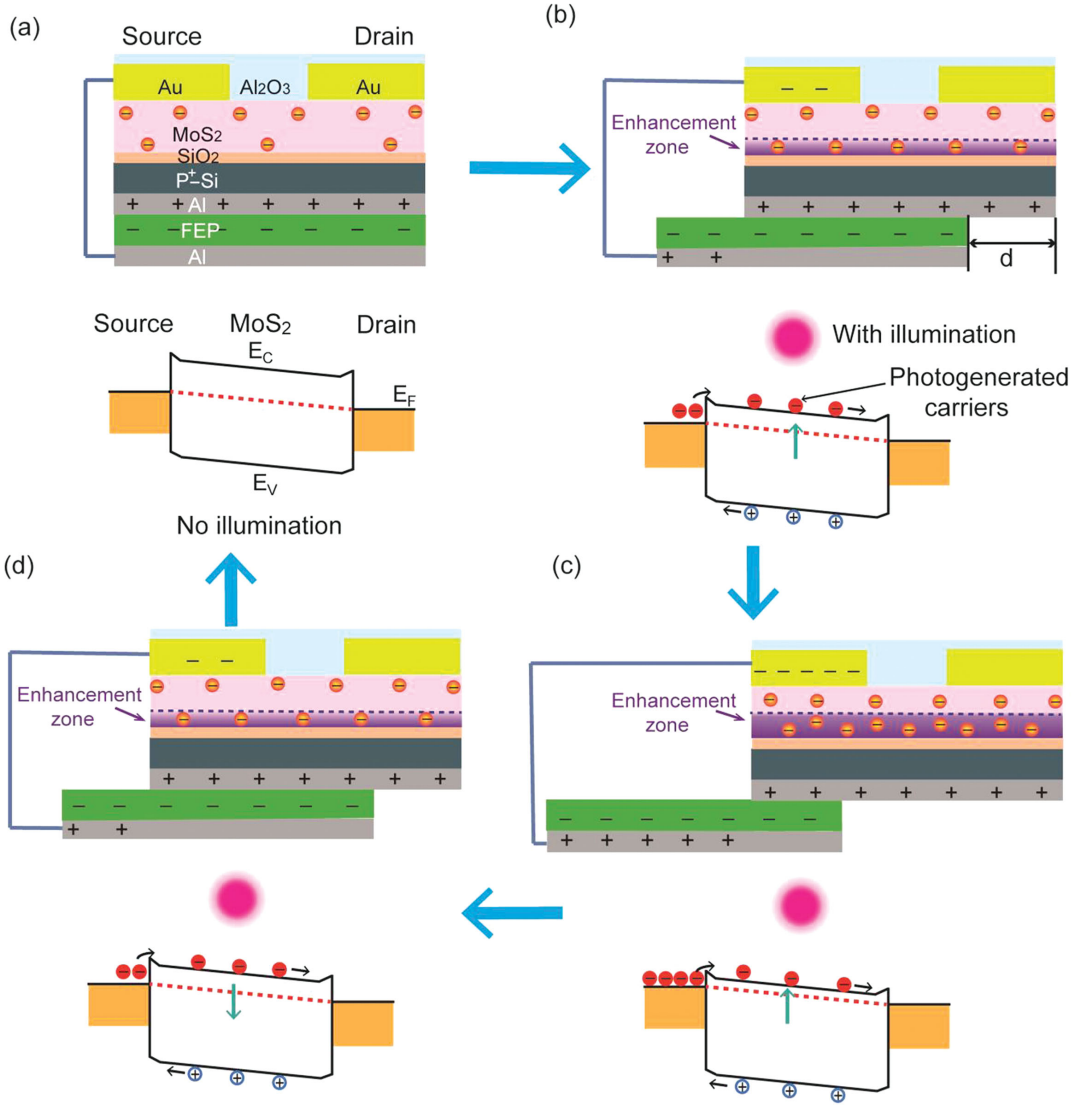
Working principle of the MoS_2_ tribotronic phototransistor. a) Initial state of the MoS_2_ tribotronic phototransistor at a bias voltage without illumination and sliding distance. b–d) The schematic interpretation and corresponding band diagrams of the MoS_2_ tribotronic phototransistor at different sliding distances with illumination.

The working principle of the MoS_2_ tribotronic phototransistor is based on the coupling effects of triboelectrification, electrostatic induction, and field effect, which is schematically shown in **Figure** [qv: **5**]. In the original state of Figure 5a, a drain voltage is applied without illumination on the device. The aluminum friction layer on the back of the device and the whole FEP friction layer fully overlap and intimately contact with each other. Owing to the different triboelectric polarity of the FEP and aluminum, the electrons are injected from the aluminum friction layer into the surface of the FEP, giving rise to the FEP film with negative charges and the aluminum friction layer with equal positive charges in the saturated state. As shown in the energy band diagram, owing to the different work function of the semiconductor and the metal, the equilibrium state forms upon the contact between Au/Cr and MoS_2_, forming a new quasi‐Fermi level. At this moment, the gate voltage is zero and conduction channel width is not influenced because the negative and positive charges on the surface of the two friction layers are fully balanced. When the FEP friction layer with the negatively charged surface slides with the aluminum friction layer by an external force (Figure 5b), some triboelectric charges lose constraint due to the decrease in the contact surface area, which will generate an electric field parallel to the sliding direction, and induce the electron flow from the aluminum electrode on the bottom of the FEP film to the source electrode. Consequently, an inner electric field voltage across the gate and source electrode is formed and an enhancement zone is formed in the n‐type MoS_2_ channel, which is similar to applying a positive voltage on the back gate of the MoS_2_ phototransistor. At the same time, the Femi level of the semiconducting MoS_2_ will be increased due to the inner gate voltage. The energy bands of the MoS_2_ will bend downwards and the barrier height between the electrode and the MoS_2_ will be lowered, giving rise to an increased photogenerated carriers passing through the channel under illumination.^4^, ^5^, ^6^ With the continuing increase of the sliding distance of the bottom FEP friction layer, as shown in Figure 5c, more photogenerated charges can be separated and efficiently passing through the channel, producing a higher photocurrent.

Thereafter, when the bottom FEP friction layer slides backward (Figure 5d), the charged surfaces begin to get in contact again and the electrons flow back from the drain electrode to the aluminum electrode on the bottom of the FEP film, resulting in decrease of the induced positive gate voltage and photocurrent. Once the two friction layers overlap again, there will be no separated charges, and the MoS_2_ phototransistor returns to the original state in Figure 5a. In this entire cycle, it is demonstrated that the inner gate voltage can be created and effectively tune the photocurrent of the MoS_2_ phototransistor by the sliding distance between the two friction layers, which has the same effect as applying an external gate voltage.

## 3 Conclusion

In summary, a MoS_2_ tribotronic phototransistor by coupling of a MoS_2_ phototransistor and a TENG in sliding mode has been fabricated for the first time, and the tribotronic enhanced optoelectric characteristics are investigated in details. With the horizontally sliding of the two friction layers, the drain–source current is increased from 0.72 to 3.85 μA at a 5 V bias voltage without illumination in place of the traditional gate voltage. Furthermore, the photoresponsivity of the MoS_2_ phototransistor can be effectively enhanced from 221.03 to 727.87 A W^−1^ with 1 V drain voltage by the induced inner electrostatic potential of the sliding electrification. By introducing 2D material‐based photoelectronics into the new fields of tribotronics, a novel and effective way has been developed to improve the photoresponsivity of the device for photodetection, which may has important prospects in human–computer interaction, touching optoelectronics and internet of things.

## 4 Experimental Section

*Characterization*: The surface morphology of PTFE film was characterized by scanning electron microscope (SEM, FEI‐8020). A common Hg–Xe lamp (LC8‐TLSX1046C03) was used in the experiment. The diameter of beam spot is 10 mm, which illuminates the whole device. The experimental scheme is shown in Figure S7 (Supporting Information).

## Supporting information

As a service to our authors and readers, this journal provides supporting information supplied by the authors. Such materials are peer reviewed and may be re‐organized for online delivery, but are not copy‐edited or typeset. Technical support issues arising from supporting information (other than missing files) should be addressed to the authors.

Supplementary
