# Supplementary material for: Tribotronic Enhanced Photoresponsivity of a MoS2 Phototransistor
Source: Adv Sci (Weinh). 2016 Feb 18;3(6):1500419. doi: 10.1002/advs.201500419 (PMC5067630; doi:10.1002/advs.201500419)
Supplement: Supplementary file 1 — Supplementary [file ADVS-3-0c-s001.pdf]

Copyright WILEY-VCH Verlag GmbH & Co. KGaA, 69469 Weinheim, Germany, 2016.

ADVANCED SCIENCE
Open Access

Supporting Information

for *Adv. Sci.*, DOI: 10.1002/advs. 201500419

Tribotronic Enhanced Photoresponsivity of a MoS_2 Phototransistor

Yaokun Pang, Fei Xue, Longfei Wang, Jian Chen, Jianjun Luo, Tao Jiang, Chi Zhang, and Zhong Lin Wang**

Supporting Information

Tribotronic Enhanced Photo-responsivity of a MoS_2 Phototransistor

Yaokun Pang+, Fei Xue+, Longfei Wang, Jian Chen, Jianjun Luo, TaoJiang, Chi Zhang and Zhong Lin Wang**

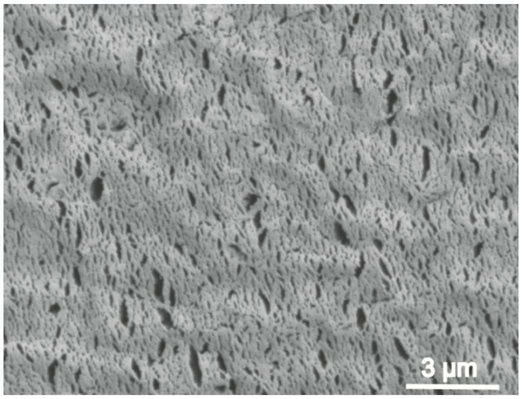

Figure S1. SEM image of the FEP surface with etched nanostructures.

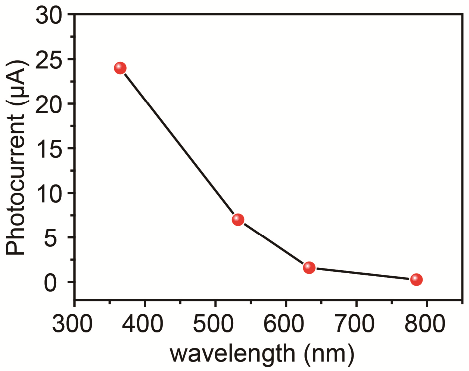

Figure S2. Photocurrent as a function of illumination wavelength.

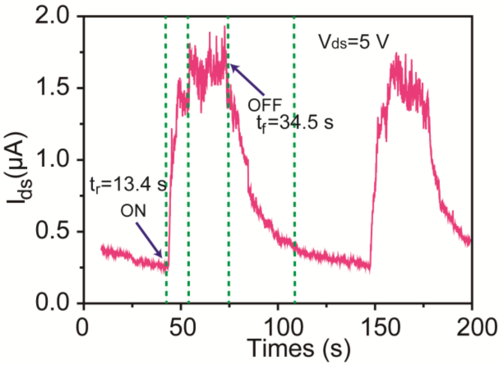

Figure S3. Response and recovery time of the MoS_2 phototransistor. The response time is 13.4 s and the recovery time is about 34.5 s.

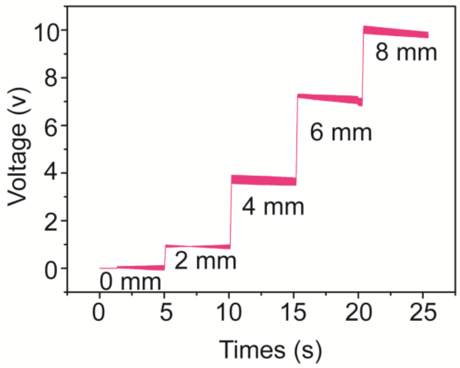

Figure S4. The relationship between the effective positive gate bias and the sliding distance.

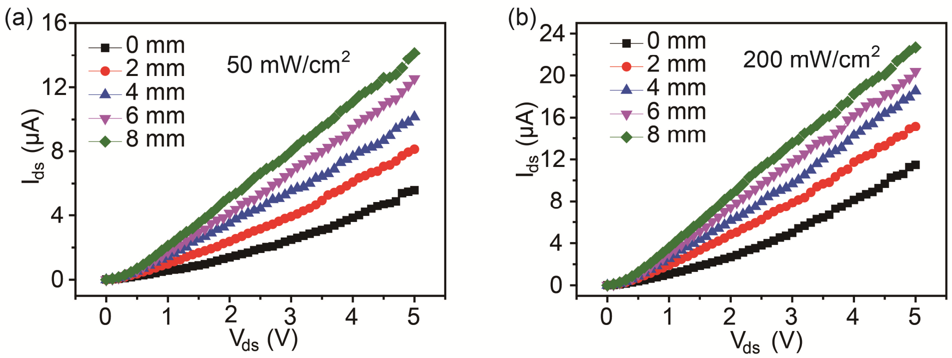

Figure S5. I_{ds}-V_{ds} curves with the different excitation intensity of 50 and 200 mW/cm^2, respectively.

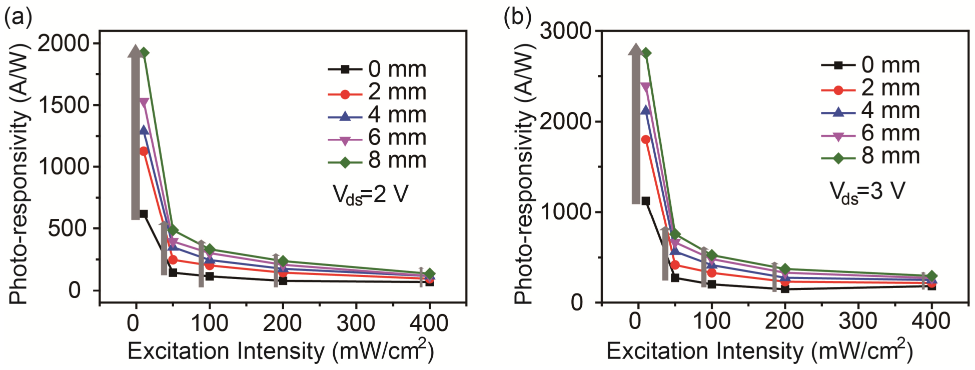

Figure S6. The relationship between the photo-responsivity and excitation intensity at different sliding distances and V_{ds}.

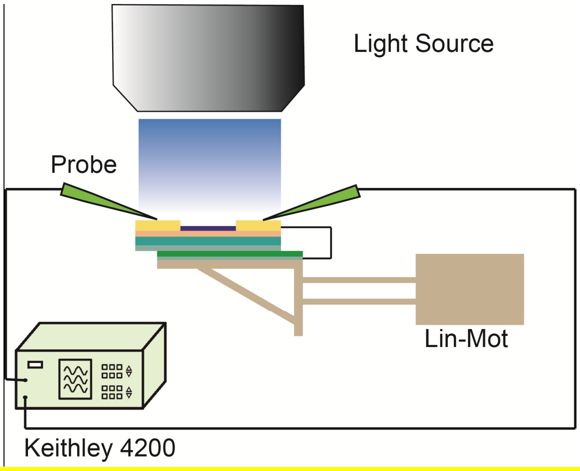

Figure S7. The experimental system for the optical-electrical characteristics of MoS_2 phototransistor.
